# White-Toothed Shrews (Genus *Crocidura*): Potential Reservoirs for Zoonotic *Leptospira* spp. and Arthropod-Borne Pathogens?

**DOI:** 10.3390/pathogens12060781

**Published:** 2023-05-30

**Authors:** Viola Haring, Jens Jacob, Bernd Walther, Martin Trost, Michael Stubbe, Katja Mertens-Scholz, Falk Melzer, Nelly Scuda, Michaela Gentil, Wolfdieter Sixl, Tanja Schäfer, Michal Stanko, Ronny Wolf, Martin Pfeffer, Rainer G. Ulrich, Anna Obiegala

**Affiliations:** 1 Institute of Novel and Emerging Infectious Diseases, Friedrich-Loeffler-Institut, Federal Research Institute for Animal Health, Südufer 10, 17493 Greifswald-Insel Riems, Germany; viola.haring@fli.de (V.H.); rainer.ulrich@fli.de (R.G.U.); 2 Institute for Epidemiology and Pathogen Diagnostics, Rodent Research, Julius Kühn-Institute, Federal Research Centre for Cultivated Plants, Toppheideweg 88, 48161 Münster, Germany; jens.jacob@julius-kuehn.de (J.J.); bernd.walther@julius-kuehn.de (B.W.); 3 Dezernat Artenschutz, Staatliche Vogelschutzwarte und CITES, Landesamt für Umweltschutz Sachsen-Anhalt, Reideburger Straße 47, 06116 Halle (Saale), Germany; martin.trost@lau.mwu.sachsen-anhalt.de; 4 Zentralmagazin Naturwissenschaftlicher Sammlungen, Martin-Luther-Universität Halle-Wittenberg, Domplatz 4, 06108 Halle (Saale), Germany; annegret.stubbe@zoologie.uni-halle.de; 5 Institute of Bacterial Infections and Zoonoses, Friedrich-Loeffler-Institut, Federal Research Institute for Animal Health, Naumburger Str. 96a, 07743 Jena, Germany; katja.mertens-scholz@fli.de (K.M.-S.); falk.melzer@fli.de (F.M.); 6 Bavarian Health and Food Safety Authority, Eggenreuther Weg 43, 91058 Erlangen, Germany; nelly.scuda@lgl.bayern.de; 7 Laboklin GmbH & Co.KG, Steubenstrasse 4, 97688 Bad Kissingen, Germany; gentil@laboklin.com; 8 Institute of Hygiene, University of Graz, 8010 Graz, Austria; wolfdieter.sixl@chello.at; 9 Wildtierhilfe Schäfer e.V., Waldstraße 275, 63071 Offenbach, Germany; info@wildtierhilfe-schaefer.de; 10 Institute of Parasitology, Slovak Academy of Sciences, Hlinkova 3, 04001 Košice, Slovakia; stankom@saske.sk; 11 Institute of Biology, Molecular Evolution and Systematics of Animals, University of Leipzig, Talstraße 33, 04103 Leipzig, Germany; rwolf@rz.uni-leipzig.de; 12 Institute of Animal Hygiene and Veterinary Public Health, University of Leipzig, An den Tierkliniken 41-43, 04103 Leipzig, Germany; pfeffer@vetmed.uni-leipzig.de

**Keywords:** shrew, reservoir, *Leptospira* spp., *Anaplasma phagocytophilum*, *Neoehrlichia mikurensis*, *Babesia* spp., *Bartonella* spp., *Coxiella burnetii*, *Brucella* spp., distribution

## Abstract

Three species of white-toothed shrews of the order Eulipotyphla are present in central Europe: the bicolored (*Crocidura leucodon*), greater (*Crocidura russula*) and lesser (*Crocidura suaveolens*) white-toothed shrews. Their precise distribution in Germany is ill-defined and little is known about them as reservoirs for zoonotic pathogens (*Leptospira* spp., *Coxiella burnetii*, *Brucella* spp., *Anaplasma phagocytophilum*, *Babesia* spp., *Neoehrlichia mikurensis* and *Bartonella* spp.). We investigated 372 *Crocidura* spp. from Germany (n = 341), Austria (n = 18), Luxembourg (n = 2) and Slovakia (n = 11). West European hedgehogs (*Erinaceus europaeus*) were added to compare the presence of pathogens in co-occurring insectivores. *Crocidura russula* were distributed mainly in western and *C. suaveolens* mainly in north-eastern Germany. *Crocidura leucodon* occurred in overlapping ranges with the other shrews. *Leptospira* spp. DNA was detected in 28/227 *C. russula* and 2/78 *C. leucodon* samples. Further characterization revealed that *Leptospira kirschneri* had a sequence type (ST) 100. *Neoehrlichia mikurensis* DNA was detected in spleen tissue from 2/213 *C. russula* samples. Hedgehogs carried DNA from *L. kirschneri* (ST 100), *L. interrogans* (ST 24), *A. phagocytophilum* and two *Bartonella* species. This study improves the knowledge of the current distribution of *Crocidura* shrews and identifies *C. russula* as carrier of *Leptospira kirschneri*. However, shrews seem to play little-to-no role in the circulation of the arthropod-borne pathogens investigated.

## 1. Introduction

Shrews are small insectivorous mammals belonging to one of the largest mammalian families, the Soricidae [1]. Currently, 448 recent species are recognised, and new species continue to be discovered [2,3,4]. The family Soricidae is divided into three subfamilies: Soricinae (red-toothed shrews), Crocidurinae (white-toothed shrews) and Myosoricinae (African white-toothed shrews) [5,6]. Representatives of the subfamily Soricinae are most abundant in the Holarctic region, while crocidurine shrews evolved, and are only present, in Eurasia and Africa [7]. In central Europe, six species of red-toothed shrews (genus *Sorex*) and three species of white-toothed shrews (genus *Crocidura*) are described [1]. They differ not only by morphological traits such as tooth colour, but also in their behaviour and ecology. *Sorex* shrews prefer cool and moist, forest-covered habitats, while *Crocidura* shrews are found in dry and arid, more open spaces and can be commensal [8,9]. The most prevalent shrew species in Germany is the common shrew (*Sorex araneus*).

The exact distribution ranges of these shrews are scarcely described, especially for white-toothed shrews. The lesser white-toothed shrew (*Crocidura suaveolens* (Pallas, 1811)) and the bicolored white-toothed shrew (*Crocidura leucodon* (Hermann, 1780)) are sympatrically found mainly in southern and eastern Europe [10]. The current distribution range of the greater white-toothed shrew (*Crocidura russula* (Hermann, 1780)) expands from northern Africa through the Iberian Peninsula and France into Germany [11]. The colonization of Ireland [12] and Great Britain [13], as well as an ongoing northward [14] and eastward [15,16] expansion of *C. russula* within Germany, have been described. In areas newly colonised by *C. russula*, competition with the smaller *C. leucodon* and *C. suaveolens* has led to their local extinction [15,16,17,18].

The role of shrews as carriers for zoonotic pathogens is still understudied [19,20], and the few available studies focused mainly on the genus *Sorex* with the detection of several different hantaviruses of unknown zoonotic potential, such as the Seewis virus and the Asikkala virus [21,22]. Shrews of the Crocidurinae subfamily are even more poorly studied, except for *C. leucodon* as a proposed reservoir for Borna Disease Virus 1 (BoDV-1; species: *Orthobornavirus bornaense*; family: *Bornaviridae*) [23,24]. Other insectivorous species, such as the West European hedgehog (*Erinaceus europaeus*, Linnaeus, 1758), are well known major carriers of *Leptospira* spp. [25] and arthropod-borne pathogens [26,27,28]. Investigation in those species has provided good insight into potential pathogens carried by shrews, as they share habitats [1].

*Leptospira* spp. are obligate extracellular bacteria belonging to the phylum Spirochaetes. They are distributed worldwide and are associated with different reservoir host species, of which small mammals are the most important [29]. The bacteria are excreted into the environment via urine and may be transmitted via contaminated water and food or via direct contact to skin lesions or conjunctivae. Clinical manifestation of an infection with *Leptospira* spp. varies from mild flu-like symptoms to severe forms such as kidney organ failure (Morbus Weil) or encephalitis [29]. Studies of *Leptospira* spp. prevalence in small mammals in Germany have mainly focused on rodents and *Sorex* shrews [30], with the occasional detection of *Leptospira kirschneri* in *C. russula* and *C. leucodon* [31,32]. Interestingly, *Leptospira alstonii* was isolated from invasive *C. russula* in Ireland, with previous isolates only originating from non-mammal hosts from China, Japan and Malaysia [33]. Little is known about the presence or prevalence of *Leptospira* spp. in lesser and bicolored white-toothed shrews.

*Anaplasma phagocytophilum*, *Babesia* spp. and *Neoehrlichia mikurensis* are tick-borne pathogens transmitted by hard ticks, mostly of the genus *Ixodes* [34], causing febrile illness in humans, especially in immunocompromised patients [35]. High prevalence rates of tick-borne pathogens were described in the common shrew [36,37], but little is known about the prevalence of these pathogens in white-toothed shrews. *Bartonella* spp., most of which are considered zoonotic [35], are Gram-negative bacteria mainly transmitted by haemophilic arthropods (fleas, ticks and lice) and can persist in erythrocytes and endothelial cells in reservoir hosts (mainly rodents, cats (*Felis catus*) and game). The detection of *Bartonella* spp. in shrews was described for *Sorex* spp. in Germany [37]. A newly described *Bartonella* strain, named *Bartonella florenciae,* was previously isolated from the spleen tissue of a *C. russula* from France [38,39].

The causative agent of “Q-fever”, *Coxiella burnetii*, is a globally distributed Gram-negative bacterium that causes infertility and abortions, mainly in ruminants (cattle, goats and sheep), and is excreted in great numbers with birth materials and, to a lesser extent in milk, faeces and urine. Farmers, veterinarians and abattoir employees are high-risk groups for infection. Numbers on reported human infections have fluctuated between 55 and 416 cases per year in Germany since 2001 [40]. Ticks (in Germany, supposedly *Dermacentor marginatus*) can shed *C. burnetii* in their faeces and transmission could potentially occur through inhalation of faecal dust rather than by the tick bite [41]. There is only limited information about the role of small mammals in the infection cycle of *C. burnetii*. A seroprevalence of 19% was previously reported for rodents in the UK [42,43]. In the vicinity of Q-fever-positive farms, seroprevalences of up to 53% in wild rats have been observed [44]. Conversely, a study on small mammals from Slovakia reported a seroprevalence of only 2.2%, while investigated *Sorex* spp. had no antibodies against *C. burnetii* [45].

*Brucella* spp. are facultative intracellular bacteria that cause brucellosis, a severe disease in animals (reproductive failure and abortion) and humans (feverish multi-organ failure). Germany is considered to be free of bovine, ovine and caprine brucellosis. To maintain this status, its potential reintroduction by wildlife should be closely monitored. However, reported human cases are increasing [46]. Several years ago, a new *Brucella* species, *Brucella microti,* was isolated from common voles in central Europe [47] and has since been detected in other wildlife [48,49]. Previous studies identified that 8% of all investigated soricine shrews [50] were *Brucella* spp.-positive, but so far no data are available on the presence of this pathogen in *Crocidura* spp. from Germany.

As data on the current distribution of greater, lesser and bicolored white-toothed shrews in Germany are incomprehensive and knowledge on their role as carriers for pathogens with zoonotic potential is limited, the objectives for this study were to (i) contribute to the current knowledge on the distribution of white-toothed shrews in Germany, (ii) detect and characterise *Leptospira* spp. in white-toothed shrews and (iii) evaluate white-toothed shrews as reservoirs for arthropod-borne pathogens and compare the findings to European hedgehogs.

## 2. Materials and Methods

### 2.1. Collection and Dissection of Shrews and Hedgehogs

**Figure 1:**
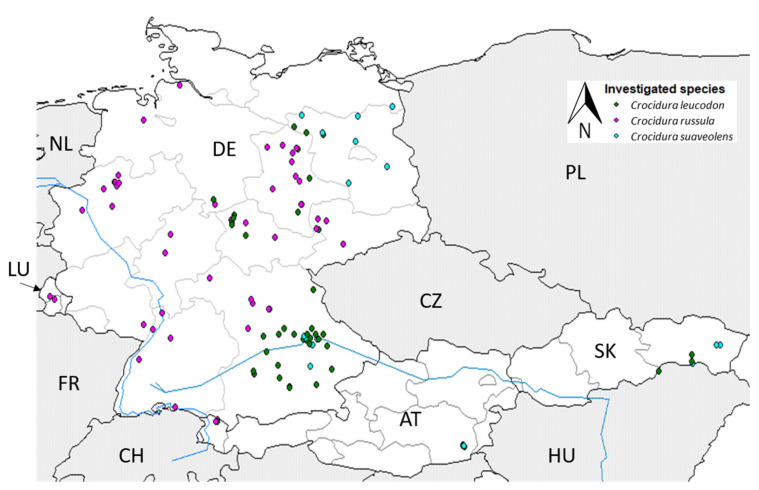
Origin of the investigated white-toothed shrews from Germany (n = 341), Luxembourg (n = 2), Austria (n = 18) and Slovakia (n = 11) based on common postal code; per trapping site, each detected species is represented by one dot. NL: the Netherlands; LU: Luxembourg; FR: France; DE: Germany; CH: Switzerland; AT: Austria; CZ: Czech Republic; PL: Poland; SK: Slovakia; HU: Hungary.

Shrews from Germany, Luxembourg, Austria and Slovakia were collected between 1999 and 2021 (Figure 1, Table S1). The majority of these originated from a citizen-science-based project, where the public was asked to send in shrews trapped by cats or found dead. Additionally, shrews were trapped as by-catch during various rodent monitoring studies and pest control measures in Germany [32,51]. European hedgehogs were collected at a rescue center in Offenbach, Germany. Information on collection date and site were recorded; the latter was defined by common postal code as it was the most precise information available for specimens from prey of cats. All animals were transported on dry ice to the laboratory and stored at −20 °C until further processing. Kidney and spleen tissues were taken during a standardised necropsy procedure [52] and stored at −20 °C. Morphological metadata on body weight and sex were taken during necropsy (Table S2).

### 2.2. Nucleic Acid Extraction

Nucleic acids were extracted from kidney and spleen tissue using a Nucleo Mag Vet Kit (Macherey & Nagel, Düren, Germany) and a KingFisher™ Flex Purification System (Thermo Fisher Scientific, Darmstadt, Germany) according to the manufacturer’s instructions.

### 2.3. Molecular Species Identification

Species identification for each shrew was performed based on the molecular analysis of the almost-complete *cytochrome b* gene and sequence comparison to GenBank entries as previously described [53].

### 2.4. Polymerase-Chain-Reaction-Based Screening for Leptospira spp. DNA

Kidney-derived DNA was screened in pools of two for the presence of *Leptospira* spp. DNA with a real-time PCR (qPCR) targeting the *lipl*32 gene (expected amplicon size: 242 base pairs, bp), encoding for an outer membrane lipoprotein [54]. Positive pools were retested for each individual, and samples with a cycle threshold (Ct) value below 41 were considered as *Leptospira*-positive. As positive control, DNA of a laboratory strain of *L. kirschneri* serovar Grippotyphosa was used [55]. Three *C. leucodon* samples were investigated previously by conventional *lipl32* gene PCR [32].

### 2.5. Multilocus Sequence Typing of Leptospira spp.

Multilocus sequence typing (MLST) of seven target genes, *glmU* (amplicon size: 650 bp), *pntA* (621 bp), *sucA* (640 bp), *tpiA* (639 bp), *pfkB* (588bp), *mreA* (791 bp) and *caiB* (650 bp), was performed for samples with a Ct value < 36 following the scheme from Boonsilp et al. [56] considering modifications as described [54].

### 2.6. Amplification and Sequencing of the secY Gene of Leptospira spp.

For samples with a Ct value > 36 or with incomplete MLST results, a conventional PCR targeting the *secY* gene (657 bp) was performed to determine *Leptospira* species as previously described [54]. As positive control, DNA of a laboratory strain of *L. interrogans* serovar Icterohaemorrhagiae was used [55].

PCR products were prepared with DNA Gel Loading Dye (6x) (Thermo Fisher Scientific, Darmstadt, Germany) for gel electrophoresis in 2% agarose, and gels were stained with HDGreen Plus DNA Stain (Intas Science Imaging Instruments GmbH, Göttingen, Germany). Amplification products were visualised by UV light using the UVP GelSolo streamlined gel documentation (Analytik Jena AG, Jena, Germany). The samples were purified for sequencing using a NucleoSpin Gel and PCR clean-up kit (Macherey-Nagel GmbH & Co. KG, Düren, Germany) as recommended by the manufacturer. The sequences were trimmed using Bionumerics v.7.6.1. (Applied Maths Inc., Austin, TX, USA) and compared to available data in GenBank with the Basic Local Alignment Search Tool (BLAST) (https://blast.ncbi.nlm.nih.gov/Blast.cgi, accessed on 7 August 2022). The obtained sequences were uploaded to GenBank (accession numbers: OQ865429–OQ865435).

### 2.7. Polymerase-Chain-Reaction-Based Screening for Arthropod-Borne Pathogens, Coxiella burnetii and Brucella spp.

The presence of *Bartonella* spp. was evaluated in individual spleen DNA samples by conventional PCR targeting the nicotinamide adenine dinucleotide hydrogen dehydrogenase (NADH) subunit (nuoG) with an amplicon size of 346 bp [57]. DNA from a cultured *B. henselae* Marseille strain was used as positive control. Positive samples were further analysed by PCR targeting the *gltA* gene (amplicon size: 378 bp) [57,58]. Positive samples were purified and sequenced commercially (Interdisziplinäres Zentrum für Klinische Forschung, Leipzig, Germany). The obtained sequences were uploaded to GenBank (accession numbers: OQ865426–OQ865428). Spleen-derived DNA pools of two individuals were screened with qPCRs for the presence of *Anaplasma phagocytophilum* DNA targeting the *msp2* (major surface protein 2) gene (amplicon size: 77 bp) [59] and *Neoehrlichia mikurensis* DNA targeting the *groEL* gene (amplicon size: 99 bp) as previously described [60]. As positive controls, we used DNA from an *A. phagocytophilum* culture and DNA from a *N. mikurensis* positive yellow-necked field mouse (*Apodemus flavicollis*) from Leipzig, Germany, that was trapped in 2016 [61], respectively. Positive pools were retested on an individual level. Spleen DNA samples in pools of three were used for the detection of *Babesia* spp. DNA by conventional PCR targeting a fragment (411–452 bp) of the *18S rRNA* gene [62]. For the detection of *Coxiella burnetii* DNA and *Brucella* spp. DNA, all individual spleen-derived DNA samples were screened using a qPCR targeting the multicopy insertion element IS1111 [63] or the *bcsp31* gene [64], respectively.

### 2.8. Statistical Analysis

All statistics were performed in the GraphPad Prism Software v. 4.0 (GraphPad Software Inc., San Diego, CA, USA). Mean prevalence and confidence intervals (95% CI) for *Leptospira* spp. were determined using the Clopper and Pearson method with an alpha value of 0.05. For the prevalence of *Leptospira* and the sex of different *Crocidura* species, Fisher’s exact test was used to test independence. Tests were considered to be significant if *p* (probability) < 0.05.

### 2.9. Generation of Maps

**Figure 2:**
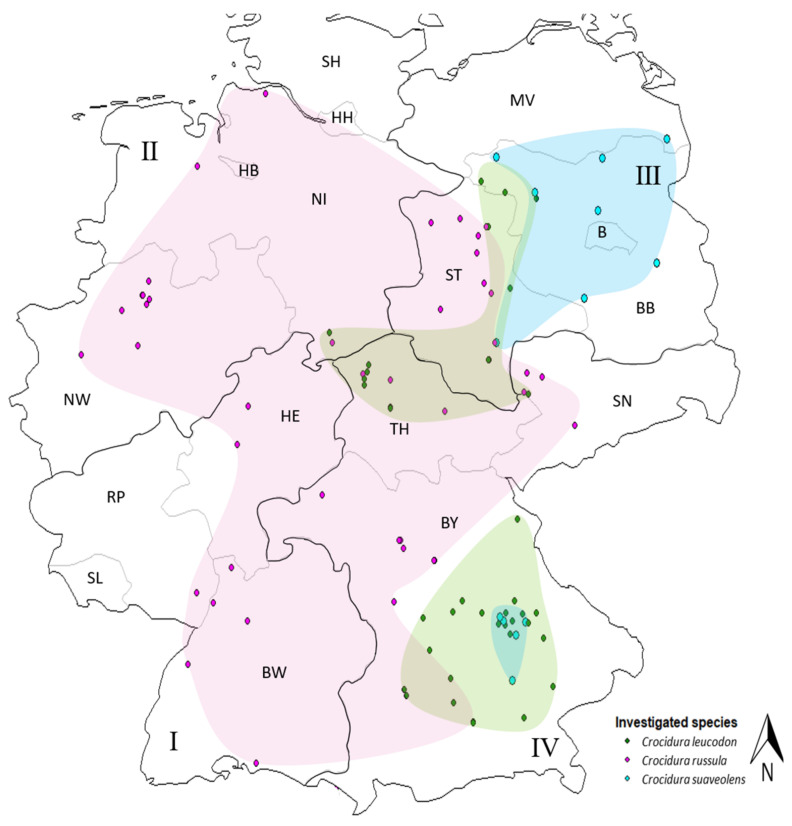
Distribution of investigated white-toothed shrews from Germany: greater white-toothed shrew (*Crocidura russula*, purple), bicolored white-toothed shrew (*Crocidura leucodon*, green), lesser white-toothed shrew (*Crocidura suaveolens*, blue); per trapping site, each detected species is represented by one dot. I Southwest: SL: Saarland, RP: Rhineland–Palatinate, BW: Baden–Wuerttemberg, HE: Hesse; II Northwest: NW: North Rhine–Westphalia, NI: Lower Saxony, HB: Bremen, HH: Hamburg; III Northeast: ST: Saxony–Anhalt, BB: Brandenburg, B: Berlin; MV: Mecklenburg–Western Pomerania; IV Southeast: BY: Bavaria, TH: Thuringia, SN: Saxony.

Maps were generated using Karten-Explorer v. 2.21 (Friedrich-Loeffler-Institut (FLI), Bundesforschungsinstitut für Tiergesundheit Copyright © 2022, Greifswald, Insel Riems, Germany). The German federal states were grouped into four regions: southwest, northwest, northeast and southeast, for the evaluation of the geographical distribution of white-toothed shrews (Figure 2).

## 3. Results

### 3.1. Distribution of White-Toothed Shrews

In total, 341 shrews were collected between 2002 and 2021 in Germany: 235 greater white-toothed shrews (68.9%; 99 males, 122 females, 14 sex not determined (s.n.d.), 83 bicolored white-toothed shrews (24.3%; 38 males, 42 females, three s.n.d.) and 23 lesser white-toothed shrews (6.7%; 12 males, 11 females) (Figure 1).

The shrews originated from the southwest (n = 9), northwest (n = 103), northeast (n = 110) and southeast (n = 118) of Germany (Figure 2). *Crocidura russula* was the most abundant species, especially in the western parts of Germany—northwest: 99% (n = 103) and southwest: 100% (n = 9). Only one *C. leucodon* (1%) was collected in the southeast of Lower Saxony close to the Harz mountain range (Table S1). In the eastern half of Germany, the situation was more diverse. All three species could be found in the northeast, with 70% *C. russula* (n = 77), 13.6% *C. leucodon* (n = 15) and 16.4% *C. suaveolens* (n = 18). *Crocidura russula* was still the predominant species in northeast Germany, but it was not collected in the state of Brandenburg (BB), which is far northeast, where mainly *C. suaveolens* was found (78.3% of all investigated *C. suaveolens*). In the southeast, especially in the south of Bavaria, *C. leucodon* was the most prominent (56.8%, n = 67), and *C. russula* (39%, n = 46) was mainly found in Franconia and further north. Of all the collected white-toothed shrews from the southeast 4.2% were *C. suaveolens* (n = 5) (Figure 2, Table S1). The species composition varied per site. The occurrence of *C. russula* and *C. leucodon* overlapped at five sites (Figure 2), and *C. leucodon* and *C. suaveolens* overlapped at four sites. *Crocidura russula* and *C. suaveolens* were only found together at one site in the northeast of Germany. We did not find all three species at the same site. A few white-toothed shrews from neighbouring countries in central Europe were included in our study: two *C. russula* from Luxembourg, two *C. russula* and one *C. leucodon* from Vorarlberg, Austria, three *C. leucodon* and twelve *C. suaveolens* from the eastern state of Steiermark, Austria, and five *C. leucodon* and six *C. suaveolens* from Slovakia (Figure 1).

### 3.2. Detection and Sequence Type Identification of Leptospira spp.

*Leptospira* spp. DNA was detected in kidney samples from 28 out of 227 *C. russula* (12.3%, 95% CI: 8.6–17.3) and three out of 81 *C. leucodon* (3.7%, 95% CI: 0.8–10.7) samples from Germany (Table 1).

All of the *C. russula* and *C. leucodon* samples from Luxembourg and Austria and all of the 22 *C. suaveolens* tested negative for the presence of *Leptospira* spp. DNA (0%, 95% CI: 0–17.6). Thus, the prevalence was significantly lower in *C. leucodon* and *C. suaveolens* compared to *C. russula* (*p* = 0.003). Out of the 28 *lipl*32 qPCR-positive *C. russula,* six were identified as *Leptospira kirschneri* by sequencing the *secY* PCR product. MLST was successful for an additional six individuals (*C. russula*) and were determined to be the same sequence type: *Leptospira kirschneri* ST 100. The sequencing of the *sec*Y PCR product of the *lipl*32 qPCR-positive *C. leucodon* was not possible, which was most likely due to the poor sample DNA quality. There was no significant difference in the prevalence between female (10.3%, 95% CI: 5.8–17.2) and male *C. russula* (14.6%, 95% CI: 8.8–23.1) (*p* = 0.337).

**Figure 3:**
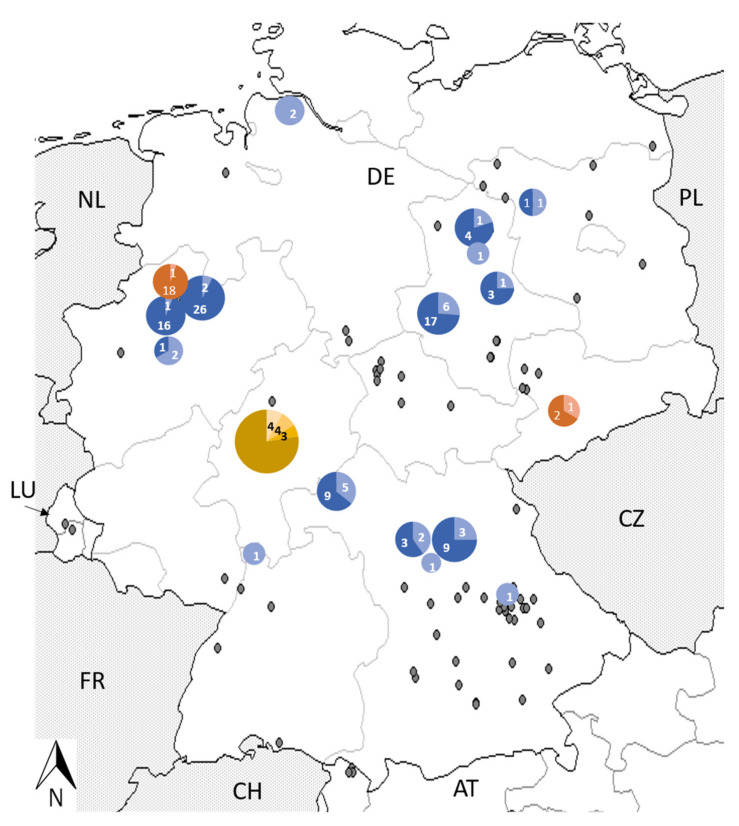
Detection of *Leptospira kirschneri* DNA (blue) and *Neoehrlichia mikurensis* DNA (orange) in white-toothed shrews. Numbers of positive individuals are indicated by a brighter colour. Trapping sites with no detection of any investigated pathogens are marked in grey. Investigations into hedgehogs are shown in yellow (four *Leptospira* spp. DNA, four *A. phagocytophilum* DNA and three *Bartonella* spp. DNA positive hedgehogs, with no co-infection).

*Leptospira* spp. DNA-positive individuals originated from 15 trapping sites from across Germany (Figure 3). The prevalence of *Leptospira kirschneri* at the different sites varied between 5.6% and 40% (mean x = 25%); sites with less than four individuals were excluded (mean x = 13; 4–33 individuals per site). The hedgehog investigation revealed that four of the 42 (9.5%, 95% CI: 3.2–22.6) animals were *lipl*32 qPCR-positive, which were further characterised as *L. kirschneri* (ST 100) and *L. interrogans* (ST 24).

### 3.3. PCR Analysis for Arthropod-Borne Pathogens, Coxiella burnetii and Brucella spp.

The PCR screening of spleen samples of 213 *C. russula*, 80 *C. leucodon* and 21 *C. suaveolens* from Germany detected *Neoehrlichia mikurensis* DNA in two female *C. russula* (0.9%, 95% CI: 0–3.6) samples, one from southeast Germany and the other one from northwest Germany (Figure 3). None of the 80 investigated *C. leucodon* (0%, 95% CI: 0–5.5) and 21 *C. suaveolens* (0%, 95% CI: 0–18.2) tested positive for *N. mikurensis* DNA. None of the investigated shrews were positive for *Babesia* spp., *A. phagocytophilum*, *Bartonella* spp., *Brucella* spp. or *C. burnetii* DNA (Table 1). The shrews from Austria and Slovakia were negative for all pathogens. The shrews from Luxembourg were not investigated due to a lack of spleen tissue. The hedgehog group indicated the presence of *A. phagocytophilum* in four of the 42 (9.5%, 95% CI: 3.2–23.6) animals. Three of the 42 (7.1%, 95% CI: 1.8–20.0) hedgehogs tested positive for *Bartonella* spp., two being typed as *B. clarridgeiae* strain 73 and one as uncultured *Bartonella* spp. None of the 42 hedgehogs tested positive for *N. mikurensis*, *Babesia* spp., *Brucella* spp. and *C. burnetii* DNA.

## 4. Discussion

### 4.1. Current Distribution of White-Toothed Shrews in Germany

The collection of 341 white-toothed shrews allowed, albeit with limitations due to the heterogenous sampling, an update on the current distribution of *Crocidura* spp. in Germany. The latest comprehensive survey on the distribution of white-toothed shrews in Germany covered only southeast Germany (Bavaria) [65] and was mainly based on the identification of skeletal remains in owl pellets. With our citizen science project, which exploited cats’ aversion to consume shrews, we were able to collect fresh carcasses to accurately identify the species using molecular techniques and to perform an initial screening of their accompanying pathogens, which allowed us to determine health risks to cats and their owners.

Over the past decades, multiple studies [9,66,67,68,69] have monitored the distribution boundaries of white-toothed shrews on local levels [14,70,71,72], describing fluctuations in total white-toothed shrew numbers [17] and uncertain boundaries. The core distribution range of *C. russula* expands from the western European countries into central Germany and is slowly expanding further east [15,73,74]. The collection of *C. russula* in our study in western and southeastern Germany coincided with the easternmost expansion into Franconia, Bavaria [65]. In regions where *C. russula* occurred, *C. russula* predominated over the other two species, which may have led to the local extinction of *C. suaveolens* as they are considered parapatric species [15,18,74]. Whether this is solely due to the size difference between the larger *C. russula* and the smaller *C. suaveolens* or due to differences in adaptations to synanthropic habitats and climate conditions, as *C. russula* copes better with drier, hotter summers, and therefore, out-competition is still under debate [15,18]. The same applies to *C. leucodon*, as *C. russula* was primarily found in former typical *C. leucodon* habitats [18,74,75,76]. The eastwards expansion of *C. russula* and the replacement of *C. leucodon* has also been observed in Switzerland [16] and Austria [8,77]. Although limited by number, we observed the same trend with *C. russula*, it being found in the northwest of Austria, while in the east of Austria so far only *C. leucodon* and *C. suaveolens* were collected. We primarily detected *C. suaveolens* in the northeastern part of Germany, supporting the westwards expansion trend described by Jentzsch and Trost [78]. *Crocidura suaveolens* were sporadically found in the southeast, but not at all in the western parts of Germany. Similarly, the absence of *C. leucodon* from the southwest was consistent with previous reports describing a decline in *C. leucodon* occurrence in the western half of Germany [68,75,76]. Information on the exact origin of an individual is needed to determine territory size and sym- and parapatry, which was not possible with our sample collection as it was greatly influenced by the cats’ behaviour. We decided to use postal codes as the smallest common spatial factor. All three species were not found together, but the co-occurrence of *C. leucodon* and *C. russula* versus *C. leucodon* and *C. suaveolens* was almost equally frequent (n = 5 vs. n = 4); however, *C. suaveolens* and *C. russula* were only collected together at one site in northeastern Germany. Between 1995 and 2010, the co-occurrence of all three species was described for east Thuringia [74] and west Saxony [18]. There are multiple possible explanations for the ongoing fluctuation and expansion of the species’ distribution ranges, including ongoing postglacial expansion [5], man-made factors due to alterations in land use and climate [79] or simply the translocation of individuals [16]. Anthropogenic movement has a great influence in the range expansion, as shrews might be transported via feed (e.g., haystacks) or soil. Once translocated, shrews easily establish new colonies [80,81,82], as seen in the introduction of the greater white-toothed shrew to Ireland, most likely due to human activity, in the early 21st century [12]. Since then, *C. russula* has expanded at a pace of 15 km/year, which is much faster than described for continental Europe.

### 4.2. Detection and Characterization of Leptospira spp. in White-Toothed Shrews

In regard to small mammals, previous studies of *Leptospira* prevalence were mainly focused on rodents and soricine shrews. Depending on the shrew species and geographic region, previous studies describe a mean *Leptospira* prevalence of 3.0% (range 0–3.4%; crowned shrew, *Sorex coronatus*), 6.8% (range 0–21.1%; pygmy shrew, *Sorex minutus*) and 15.5% (range 0–23.5%; common shrew, *Sorex araneus*) [30].

The current knowledge on *Leptospira* in crocidurine shrews in central Europe is scarce. *Leptospira* spp. was detected in *C. russula* already in the 1970s [83]. In Germany, *Leptospira kirschneri* was found in *Crocidura russula* [84] and *Crocidura leucodon* [32], but no further sequence typing was performed. Here, we detected *Leptospira kirschneri* in 28 *C. russula* and two *C. leucodon* with a mean prevalence of 25% (5.6–40%) at 15 trapping sites. *Leptospira* spp. was irregularly distributed in Germany, as demonstrated by its absence in white-toothed shrews from Saxony (this study, [85]). The irregular distribution and broad variation in the prevalence per trapping site might be caused by a biased sample size per site and the geographic origin of the samples. Water and moist areas play an important role in the maintenance and spread of *Leptospira* spp. outside their animal hosts [29]; crocidurine shrews prefer more open, arid habitats, which might explain the lower *Leptospira* spp. prevalence compared to *Sorex* spp. and rodents. The observed difference in prevalence between *C. russula* and *C. leucodon* could be due to the differences in habitat use between the species. *Crocidura russula* is a range-expanding invader [86] and may therefore have a higher exposure to *Leptospira*. Unfortunately, a comparison of the exact habitat use between the shrew species was not possible due to our sampling method. Although *Leptospira kirschneri* has been described as the most abundant genomospecies in small mammals, Jeske et al. [32] detected *Leptospira borgpetersenii* in sympatric rodents from trapping sites, where *L. kirschneri* was found in *C. leucodon*. Interestingly, the investigated hedgehogs carried two *Leptospira* species, *L. kirschneri* ST 100 and *Leptospira interrogans* ST 24, with the latter one commonly found in forest-dwelling rodents such as yellow-necked field mice and wood mice (*Apodemus sylvaticus*) [30].

MLST allowed us to determine the ST of *Leptospira* spp., and it is widely used to evaluate the spread of a specific pathogen within a population to distinguish detection in maintenance hosts from spill-over and host-switch events. In small mammal populations, different sequence types are seen within the same species and the same ST in different animal species. Common shrews from various locations in Germany have been shown to carry *Leptospira kirschneri* of two different sequence types (ST 110, ST 136) as well as *Leptospira borgpetersenii* of ST 146 [30]. *Leptospira kirschneri* ST 110 is strongly associated with voles of the genus *Microtus* and is the most common source of leptospirosis outbreaks in strawberry pickers in Germany [30]. Interestingly, we found only a single *Leptospira kirschneri* ST (ST 100) in all the *C. russula* samples from the different trapping sites across Germany, suggesting a possible host species specificity and may identify *C. russula* as maintenance host rather than spill-over host. However, this ST was also found in a European hedgehog (this study) and was previously isolated from a Portuguese house mouse (*Mus musculus*) [87]. This ST has been associated to the serovar Mozdok, a serovar that is widely distributed in small mammals (mainly *Apodemus agrarius*) in central Europe [88], which causes canine leptospirosis [89] and is also associated with human infections [90]. Further investigations on sympatric small mammals from the same trapping sites are needed to determine how widespread ST 100 is within the small mammal community. Unfortunately, for the publicly available ST 100 isolate (*Leptospira* isolate 15-LE00367-0 [91]) from Germany, the host species and its precise origin in Lower Saxony, Germany, is not specified.

### 4.3. Identification of White-Toothed Shrews as Reservoirs for Arthropod-Borne Pathogens

A high prevalence of tick-borne pathogens has been described for common shrews [36,37], but little is known about the prevalence of these pathogens in white-toothed shrews. A comparable study from Spain found *A. phagocytophilum* in one of six *C. russula* samples [92], whereas a previous study from Germany did not detect *A. phagocytophilum*, *Babesia* spp. and *N. mikurensis* in any *C. russula* sample [60]. Even though our sample size (n = 372) was much larger than that of previous studies (n = 4), we still did not detect *A. phagocytophilum* in any white-toothed shrew. *Anaplasma phagocytophilum* is present in the small mammal community in Germany, as confirmed here by the prevalence of about 10% in European hedgehogs (this study, [27]) and in crowned shrews and bank voles (*Clethrionomys glareolus*) [37]. We detected *N. mikurensis* DNA in two *C. russula* samples at different urban sites in northwestern and southeastern Germany, a finding that seems to be in contradiction to the assumption of previous studies that insectivores do not play a role in the transmission and maintenance of *N. mikurensis* [93]. The detection and further characterization of *Bartonella* spp. from soricine shrews in Germany revealed host-specific *Bartonella taylorii*-associated strains [37,94]. So far, *Bartonella* spp. have only been detected in *Crocidura* spp. outside of Germany [95,96], e.g., the detection of the new species *Bartonella refiksaydamii* in the blood of a lesser white-toothed shrew from northwestern Turkey by Celebi et al. [97]. In this study, we did not detect *Bartonella* spp. DNA in any of the white-toothed shrews, but we identified the *Bartonella clarridgeiae* strain 73 and an “uncultured *Bartonella* spp.” in the hedgehogs. *Bartonella clarridgeiae* is commonly present in cats [38], is transmitted by cat fleas (*Ctenocephalides felis*) and was once found in an asymptomatic blood donor in Brazil [98]. The role of small mammals and shrews in particular for the transmission of *Babesia* spp. and *Coxiella burnetii* is ill-defined. In our study, we did not detect *Babesia* spp. DNA in any of the crocidurine shrews or hedgehogs, even though Bown et al. [36] reported a *Babesia microti* prevalence of 30.3% in common shrews occupying the same habitat as field voles (30.4% *B. microti*-prevalence). Despite reports of a high seroprevalence for *C. burnetii* in rodents [42], all of the insectivores tested here were negative according to the PCR analysis. Assuming that small mammals are exposed to *C. burnetii*, shrews and hedgehogs do not seem to play a role as reservoirs. Fleas collected from *C. suaveolens* were tested for the presence of *C. burnetii* and rickettsiae, but they did not contain any respective DNA [99]. Previous detection of *Brucella* spp. in soricine shrews [50] could not be demonstrated for crocidurine shrews, as all of the insectivores tested here were negative.

Little is known about ectoparasites on shrews, but a white-toothed-shrew specific “ectoparasite milieu” [99,100], reducing the possible transmission of arthropod-borne pathogens from other (small mammal) species, might be an explanation for the observed low pathogen prevalence. Even though different life stages of *Ixodes ricinus* and *Dermacentor reticulatus* could be collected from *C. leucodon* and *C. suaveolens* trapped in Slovakia, the numbers were much lower than those from sympatric rodent species [101].

## 5. Conclusions

This study provides an update on the current distribution of white-toothed shrews in Germany. Altogether, white-toothed shrews seem to play a minor role in the transmission of *Leptospira* spp. and arthropod-borne pathogens. However, our study was limited by its sample size and sampling approach, heavily relying on the cooperation of the public. In the future, a more systematic and longitudinal study, ideally in a One Health setting, is needed to evaluate the potential infection risks of shrews and hedgehogs. The short life expectancy and high turnover rate of local shrew populations, including frequent extinction and fast recolonization events as described for *C. russula* [82], potentially influencing pathogen persistence in shrew communities, should be taken into account.

## Figures and Tables

**Table 1:** Results for the detection of *Leptospira* spp. with *lipl*32-qPCR in kidney tissue and *Neoehrlichia mikurensis* (*groEL*-qPCR), *Anaplasma phagocytophilum* (*msp*2-qPCR) and *Coxiella burnetii* (multicopy IS1111 element-qPCR), *Brucella* spp. (*bcsp31*-qPCR) and conventional PCR results for the detection of *Babesia* spp. (*18S* rRNA) and *Bartonella* spp. (*nuoG-*PCR) in spleen tissue of white-toothed shrews from Germany collected between 2002–2021.

Species	Number of *Leptospira* DNA-Positive/Total Number of Tested Individuals (Percentage, 95% CI *)	Number of *N. mikurensis* DNA-Positive/Total Number of Tested Individuals (Percentage, 95% CI *)	Number of *A. phagocytophilum, C. burnetii, Brucella* spp., *Babesia* spp. and *Bartonella* spp. DNA-Positive/Total Number of Tested Individuals (Percentage, 95% CI *)
Greater white-toothed shrew *** (Crocidura russula)	28/227 (12.3%, 8.6–17.3)	2/213 (0.9%, 0–3.6)	0/213 (0%, 0–2.1)
Bicoloured white-toothed shrew *** (Crocidura leucodon)	3/81 ** (3.7%, 0.8–10.7)	0/80 (0%, 0–5.5)	0/80 (0%, 0–5.5)
Lesser white-toothed shrew *** (Crocidura suaveolens)	0/22 (0%, 0–17.6)	0/21 (0%, 0–18.2)	0/21 (0%, 0–18.2)

* CI: confidence interval. ** including three *C. leucodon* previously investigated by Jeske et al. [32] *** *C. leucodon*, *C. russula* and *C. suaveolens* from Luxembourg, Austria and Slovakia tested negative for all investigated pathogens.

## Data Availability

All data are presented within the manuscript and its Supplementary Materials. Sequence data were uploaded to GenBank (accession numbers: OQ865426-OQ865435).

## Supplementary Materials

The following supporting information can be downloaded at: https://www.mdpi.com/article/10.3390/pathogens12060781/s1, Table S1: Information on the origin of investigated shrews.; Table S2: Metadata on investigated white-toothed shrews and hedgehogs.

## References

[1] Wilson D.E., Mittermaier R.A. (2017). Handbook of the Mammals of the World: Volume 8. Insectivores, Sloths and Colugos.

[2] Esselstyn J.A., Maharadatunkamsi, Achmadi A.S., Siler C.D., Evans B.J. (2013). Carving out turf in a biodiversity hotspot: Multiple, previously unrecognized shrew species co-occur on Java Island, Indonesia. Mol. Ecol..

[3] Hutterer R., Balete D.S., Giarla T.C., Heaney L.R., Esselstyn J.A. (2018). A new genus and species of shrew (Mammalia: Soricidae) from Palawan Island, Philippines. J. Mammal..

[4] Esselstyn J.A., Achmadi A.S., Handika H., Swanson M.T., Giarla T.C., Rowe K.C. (2021). Fourteen New, Endemic Species of Shrew (Genus *Crocidura*) from Sulawesi Reveal a Spectacular Island Radiation. Bull. Am. Mus. Nat. Hist..

[5] Dubey S., Zaitsev M., Cosson J.-F., Abdukadier A., Vogel P. (2006). Pliocene and Pleistocene diversification and multiple refugia in a Eurasian shrew (*Crocidura suaveolens* group). Mol. Phylogenet. Evol..

[6] Dubey S., Salamin N., Ohdachi S.D., Barrière P., Vogel P. (2007). Molecular phylogenetics of shrews (Mammalia: Soricidae) reveal timing of transcontinental colonizations. Mol. Phylogenet. Evol..

[7] Dubey S., Salamin N., Ruedi M., Barrière P., Colyn M., Vogel P. (2008). Biogeographic origin and radiation of the Old World crocidurine shrews (Mammalia: Soricidae) inferred from mitochondrial and nuclear genes. Mol. Phylogenet. Evol..

[8] Spitzenberger F. (1985). Die Weißzahnspitzmäuse (Crocidurinae) Österreichs: Mammalia austriaca 8 (Mammalia, Insectivora). Mitt. Abt. Zool..

[9] Kraft R., Klemmer W., Malec F. (2010). Kleinsäugernachweise aus dem Südlichen Oberpfälzer Wald und angrenzenden Gebieten (Ostbayern). Säugetierkundliche Inf. Jena.

[10] Mitchell-Jones A.G., Amori G., Bogdanowicz W., Krystufek B., Reijnders P.J., Spitzenberger F., Stubbe M., Thissen J.B., Vohralik V., Zima J. (1999). The Atlas of European Mammals.

[11] Cosson J.-F., Hutterer R., Libois R., Sarà M., Taberlet P., Vogel P. (2005). Phylogeographical footprints of the Strait of Gibraltar and Quaternary climatic fluctuations in the western Mediterranean: A case study with the greater white-toothed shrew, *Crocidura russula* (Mammalia: Soricidae). Mol. Ecol..

[12] Tosh D.G., Lusby J., Montgomery W.I., O‘Halloran J. (2008). First record of greater white-toothed shrew *Crocidura russula* in Ireland. Mammal Rev..

[13] Bond I.F., Gilford E., McDevitt A.D., Young M.A., Coomber F.G. (2022). First records of the greater white-toothed shrew *Crocidura russula* from Great Britain. Mammal Commun..

[14] Borkenhagen P. (1995). First record of the Greater white-toothed shrew (*Crocidura russula*) in Schleswig-Holstein, Northern Germany. Faun-Oekol-Mitt.

[15] Kraft R. (2000). Ehemalige und aktuelle Verbreitung von Hausspitzmaus, *Crocidura russula* (Hermann, 1780) und Gartenspitzmaus, *Crocidura suaveolens* (Pallas, 1811), in Bayern. Bonn.-Zool.-Beitraege.

[16] Vogel P., Jutzeler S., Rulence B., Reutter B.A. (2002). Range expansion of the greater white-toothed shrew *Crocidura russula* in Switzerland results in local extinction of the bicolored white-toothed shrew *C. leucodon*. Acta Theriol..

[17] Lehmann E.v., Brücher H. (1997). Zum Rückgang der Feld- und der Hausspitzmaus (*Crocidura leucodon* und *russula*) in Westeuropa. Bonn.-Zool.-Beitraege.

[18] Wolf R. (2010). Bestandsveränderungen und Arealverschiebungen bei den Wimperspitzmäusen (*Crocidura* Wagler, 1832) zwischen Wurzen und Grimma, Nordwestsachsen. Mitt. Für Sächsische Säugetierfreunde.

[19] Zhang X.-A., Li H., Jiang F.-C., Zhu F., Zhang Y.-F., Chen J.-J., Tan C.-W., Anderson D.E., Fan H., Dong L.-Y. (2022). A Zoonotic Henipavirus in Febrile Patients in China. N. Engl. J. Med..

[20] Johne R., Tausch S.H., Schilling-Loeffler K., Ulrich R.G. (2022). Genome sequence analysis of a novel rotavirus strain indicates a broad genetic diversity of rotavirus A in shrews. Infect. Genet. Evol..

[21] Schlegel M., Radosa L., Rosenfeld U.M., Schmidt S., Triebenbacher C., Löhr P.-W., Fuchs D., Heroldová M., Jánová E., Stanko M. (2012). Broad geographical distribution and high genetic diversity of shrew-borne Seewis hantavirus in Central Europe. Virus Genes.

[22] Radosa L., Schlegel M., Gebauer P., Ansorge H., Heroldová M., Jánová E., Stanko M., Mošanský L., Fričová J., Pejčoch M. (2013). Detection of shrew-borne hantavirus in Eurasian pygmy shrew (*Sorex minutus*) in Central Europe. Infect. Genet. Evol..

[23] Hilbe M., Herrsche R., Kolodziejek J., Nowotny N., Zlinszky K., Ehrensperger F. (2006). Shrews as reservoir hosts of borna disease virus. Emerg. Infect. Dis..

[24] Dürrwald R., Kolodziejek J., Weissenböck H., Nowotny N. (2014). The bicolored white-toothed shrew *Crocidura leucodon* (HERMANN 1780) is an indigenous host of mammalian Borna disease virus. PLoS ONE.

[25] Ayral F., Djelouadji Z., Raton V., Zilber A.-L., Gasqui P., Faure E., Baurier F., Vourc’h G., Kodjo A., Combes B. (2016). Hedgehogs and Mustelid Species: Major Carriers of Pathogenic *Leptospira*, a Survey in 28 Animal Species in France (20122015). PLoS ONE.

[26] Jahfari S., Ruyts S.C., Frazer-Mendelewska E., Jaarsma R., Verheyen K., Sprong H. (2017). Melting pot of tick-borne zoonoses: The European hedgehog contributes to the maintenance of various tick-borne diseases in natural cycles urban and suburban areas. Parasit. Vectors.

[27] Skuballa J., Petney T., Pfäffle M., Taraschewski H. (2010). Molecular detection of *Anaplasma phagocytophilum* in the European hedgehog (*Erinaceus europaeus*) and its ticks. Vector Borne Zoonotic Dis..

[28] Majerová K., Gutiérrez R., Fonville M., Hönig V., Papežík P., Hofmannová L., Lesiczka P.M., Nachum-Biala Y., Růžek D., Sprong H. (2021). Hedgehogs and Squirrels as Hosts of Zoonotic *Bartonella* Species. Pathogens.

[29] Bharti A.R., Nally J.E., Ricaldi J.N., Matthias M.A., Diaz M.M., Lovett M.A., Levett P.N., Gilman R.H., Willig M.R., Gotuzzo E. (2003). Leptospirosis: A zoonotic disease of global importance. Lancet Infect. Dis..

[30] Fischer S., Mayer-Scholl A., Imholt C., Spierling N.G., Heuser E., Schmidt S., Reil D., Rosenfeld U.M., Jacob J., Nöckler K. (2018). *Leptospira* Genomospecies and Sequence Type Prevalence in Small Mammal Populations in Germany. Vector Borne Zoonotic Dis..

[31] Mayer-Scholl A., Hammerl J.A., Schmidt S., Ulrich R.G., Pfeffer M., Woll D., Scholz H.C., Thomas A., Nöckler K. (2014). *Leptospira* spp. in rodents and shrews in Germany. Int. J. Environ. Res. Public Health.

[32] Jeske K., Jacob J., Drewes S., Pfeffer M., Heckel G., Ulrich R.G., Imholt C. (2021). Hantavirus-*Leptospira* coinfections in small mammals from central Germany. Epidemiol. Infect..

[33] Nally J.E., Arent Z., Bayles D.O., Hornsby R.L., Gilmore C., Regan S., McDevitt A.D., Yearsley J., Fanning S., McMahon B.J. (2016). Emerging Infectious Disease Implications of Invasive Mammalian Species: The Greater White-Toothed Shrew (*Crocidura russula*) Is Associated With a Novel Serovar of Pathogenic *Leptospira* in Ireland. PLoS Negl. Trop. Dis..

[34] Rizzoli A., Silaghi C., Obiegala A., Rudolf I., Hubálek Z., Földvári G., Plantard O., Vayssier-Taussat M., Bonnet S., Spitalská E. (2014). *Ixodes ricinus* and Its Transmitted Pathogens in Urban and Peri-Urban Areas in Europe: New Hazards and Relevance for Public Health. Front. Public Health.

[35] Rar V., Golovljova I. (2011). *Anaplasma*, *Ehrlichia*, and “*Candidatus* Neoehrlichia” bacteria: Pathogenicity, biodiversity, and molecular genetic characteristics, a review. Infect. Genet. Evol..

[36] Bown K.J., Lambin X., Telford G., Heyder-Bruckner D., Ogden N.H., Birtles R.J. (2011). The common shrew (*Sorex araneus*): A neglected host of tick-borne infections?. Vector Borne Zoonotic Dis..

[37] Obiegala A., Jeske K., Augustin M., Król N., Fischer S., Mertens-Scholz K., Imholt C., Suchomel J., Heroldova M., Tomaso H. (2019). Highly prevalent bartonellae and other vector-borne pathogens in small mammal species from the Czech Republic and Germany. Parasit. Vectors.

[38] Krügel M., Król N., Kempf V.A.J., Pfeffer M., Obiegala A. (2022). Emerging rodent-associated *Bartonella*: A threat for human health?. Parasit. Vectors.

[39] Mediannikov O., El Karkouri K., Robert C., Fournier P.-E., Raoult D. (2013). Non-contiguous finished genome sequence and description of *Bartonella florenciae* sp. nov. Stand. Genom. Sci..

[40] Robert Koch-Institut (2021). Infektionsepidemiologisches Jahrbuch meldepflichtiger Krankheiten für 2020. Berl. Ger..

[41] Körner S., Makert G.R., Ulbert S., Pfeffer M., Mertens-Scholz K. (2021). The Prevalence of *Coxiella burnetii* in Hard Ticks in Europe and Their Role in Q Fever Transmission Revisited-A Systematic Review. Front. Vet. Sci..

[42] Meredith A.L., Cleaveland S.C., Denwood M.J., Brown J.K., Shaw D.J. (2015). *Coxiella burnetii* (Q-Fever) Seroprevalence in Prey and Predators in the United Kingdom: Evaluation of Infection in Wild Rodents, Foxes and Domestic Cats Using a Modified ELISA. Transbound. Emerg. Dis..

[43] Webster J.P., Lloyd G., Macdonald D.W. (1995). Q fever (*Coxiella burnetii*) reservoir in wild brown rat (*Rattus norvegicus*) populations in the UK. Parasitology.

[44] Reusken C., van der Plaats R., Opsteegh M., de Bruin A., Swart A. (2011). *Coxiella burnetii* (Q fever) in *Rattus norvegicus* and *Rattus rattus* at livestock farms and urban locations in the Netherlands; could *Rattus* spp. represent reservoirs for (re)introduction?. Prev. Vet. Med..

[45] Rehácek J., Zupancicová M., Ac P., Tarasevic I.V., Jablonskaja V.A., Pospísil R., Baloghovă D. (1976). Ricettsioses studies. 2. Natural foci of rickettsioses in east Slovakia. Bull. World Health Organ..

[46] Enkelmann J., Stark K., Faber M. (2020). Epidemiological trends of notified human brucellosis in Germany, 2006–2018. Int. J. Infect. Dis..

[47] Scholz H.C., Hubalek Z., Sedlácek I., Vergnaud G., Tomaso H., Al Dahouk S., Melzer F., Kämpfer P., Neubauer H., Cloeckaert A. (2008). *Brucella microti* sp. nov., isolated from the common vole *Microtus arvalis*. Int. J. Syst. Evol. Microbiol..

[48] Scholz H.C., Hofer E., Vergnaud G., Le Fleche P., Whatmore A.M., Al Dahouk S., Pfeffer M., Krüger M., Cloeckaert A., Tomaso H. (2009). Isolation of *Brucella microti* from mandibular lymph nodes of red foxes, *Vulpes vulpes*, in lower Austria. Vector Borne Zoonotic Dis..

[49] Rónai Z., Kreizinger Z., Dán Á., Drees K., Foster J.T., Bányai K., Marton S., Szeredi L., Jánosi S., Gyuranecz M. (2015). First isolation and characterization of *Brucella microti* from wild boar. BMC Vet. Res..

[50] Hammerl J.A., Ulrich R.G., Imholt C., Scholz H.C., Jacob J., Kratzmann N., Nöckler K., Al Dahouk S. (2017). Molecular Survey on Brucellosis in Rodents and Shrews—Natural Reservoirs of Novel *Brucella* Species in Germany?. Transbound. Emerg. Dis..

[51] Schulze V., Große R., Fürstenau J., Forth L.F., Ebinger A., Richter M.T., Tappe D., Mertsch T., Klose K., Schlottau K. (2020). Borna disease outbreak with high mortality in an alpaca herd in a previously unreported endemic area in Germany. Transbound. Emerg. Dis..

[52] Ulrich R.G., Schmidt-Chanasit J., Schlegel M., Jacob J., Pelz H.-J., Mertens M., Wenk M., Büchner T., Masur D., Sevke K. (2008). Network “Rodent-borne pathogens” in Germany: Longitudinal studies on the geographical distribution and prevalence of hantavirus infections. Parasitol. Res..

[53] Schlegel M., Ali H.S., Stieger N., Groschup M.H., Wolf R., Ulrich R.G. (2012). Molecular identification of small mammal species using novel *Cytochrome b* gene-derived degenerated primers. Biochem. Genet..

[54] Schmidt E., Obiegala A., Imholt C., Drewes S., Saathoff M., Freise J., Runge M., Jacob J., Mayer-Scholl A., Ulrich R.G. (2021). Influence of Season, Population and Individual Characteristics on the Prevalence of *Leptospira* spp. in Bank Voles in North-West Germany. Biology.

[55] Nau L.H., Obiegala A., Król N., Mayer-Scholl A., Pfeffer M. (2020). Survival time of *Leptospira kirschneri* serovar Grippotyphosa under different environmental conditions. PLoS ONE.

[56] Boonsilp S., Thaipadungpanit J., Amornchai P., Wuthiekanun V., Bailey M.S., Holden M.T.G., Zhang C., Jiang X., Koizumi N., Taylor K. (2013). A single multilocus sequence typing (MLST) scheme for seven pathogenic *Leptospira* species. PLoS Negl. Trop. Dis..

[57] Norman A.F., Regnery R., Jameson P., Greene C., Krause D.C. (1995). Differentiation of *Bartonella*-like isolates at the species level by PCR-restriction fragment length polymorphism in the citrate synthase gene. J. Clin. Microbiol..

[58] Kosoy M.Y., Regnery R.L., Tzianabos T., Marston E.L., Jones D.C., Green D., Maupin G.O., Olson J.G., Childs J.E. (1997). Distribution, diversity, and host specificity of *Bartonella* in rodents from the Southeastern United States. Am. J. Trop. Med. Hyg..

[59] Silaghi C., Liebisch G., Pfister K. (2011). Genetic variants of *Anaplasma phagocytophilum* from 14 equine granulocytic anaplasmosis cases. Parasit. Vectors.

[60] Silaghi C., Woll D., Mahling M., Pfister K., Pfeffer M. (2012). *Candidatus* Neoehrlichia mikurensis in rodents in an area with sympatric existence of the hard ticks *Ixodes ricinus* and *Dermacentor reticulatus*, Germany. Parasit. Vectors.

[61] Galfsky D., Król N., Pfeffer M., Obiegala A. (2019). Long-term trends of tick-borne pathogens in regard to small mammal and tick populations from Saxony, Germany. Parasit. Vectors.

[62] Obiegala A., Heuser E., Ryll R., Imholt C., Fürst J., Prautsch L.-M., Plenge-Bönig A., Ulrich R.G., Pfeffer M. (2019). Norway and black rats in Europe: Potential reservoirs for zoonotic arthropod-borne pathogens?. Pest Manag. Sci..

[63] Klee S.R., Tyczka J., Ellerbrok H., Franz T., Linke S., Baljer G., Appel B. (2006). Highly sensitive real-time PCR for specific detection and quantification of *Coxiella burnetii*. BMC Microbiol..

[64] Probert W.S., Schrader K.N., Khuong N.Y., Bystrom S.L., Graves M.H. (2004). Real-time multiplex PCR assay for detection of *Brucella* spp., *B. abortus*, and *B. melitensis*. J. Clin. Microbiol..

[65] Kraft R. (2008). Mäuse und Spitzmäuse in Bayern: Verbreitung, Lebensraum, Bestandssituation.

[66] Niethammer J. (1980). Zur gegenwärtigen Nordgrenze von *Crocidura leucodon* in Niedersachsen. Z. Säugetierkunde.

[67] Roschen A., Hellbernd L., Nettmann H.-K. (1984). Die Verbreitung von *Crocidura russula* und *Crocidura leucodon* in der Bremer Wesermarsch. Z. Säugetierkunde.

[68] Güttinger R., Pfunder M., Wüst M. (2008). Die Verbreitung von Feldspitzmaus *Crodidura leucodon* und Hausspitzmaus *C. russula* in der Ostschweiz: Eine spezielle Situation in ihrer zoogeografischen Kontaktzone. Gall. Nat. Ges..

[69] Krämer M., Jentzsch M. (2008). Kleinsäuger-Vorkommen aus dem Raum Zeitz—Eine vergleichende Studie. Mauritiana.

[70] Dornberger W. (1990). Fund einer Hausspitzmaus (*Crocidura russula*) in Niederstetten. Faun-Und-Flor-Mitt.

[71] Klesser R., Jessen F., Ringenberg J., Preuß M., Kaiser T., Husemann M. (2021). Return of the walking dead: First verified record of the shrew *Crocidura leucodon* (Hermann, 1780) in Hamburg, Germany. EvolSyst.

[72] (2003). Nationalatlas Bundesrepublik Deutschland: Verbreitung der Säugetierarten.

[73] Frank F. (1984). Zur Arealverschiebung zwischen *Crocidura russula* und *C. leucodon* in NW-Deutschland und zum wechselseitigen Verhältnis beider Arten. Z. Säugetierkunde.

[74] Worschech K. (2010). Ehemaliges und gegenwärtiges Vorkommen der Weißzahnspitzmäuse (*Crocidura* W_AGLER_, 1832) im Altenburger Land (Thüringen) (Mammalia:Soricidae). Mauritiana.

[75] Paliocha E., Wilhelm P. (1993). Forschungsprojekt “Wildlebende Säugetiere in Baden-Württemberg” Bericht über das Forschungsvorhaben: 1. Gewöllanalyse. MAUS. Mitt. Unserer Säugetierwelt.

[76] Brünner H. (2010). Erfassung von Säugetieren im LIFE-Projektgebiet “Lebendige Rheinauen bei Karlsruhe. https://rp.baden-wuerttemberg.de/fileadmin/RP-Internet/Karlsruhe/Abteilung_5/Referat_56/Lebendige_Rheinauen/_DocumentLibraries/ErgebnisseundVortrge/Endberichte/08_leb_rhein_endb_saeuger.pdf.

[77] Spitzenberger F. (2001). Die Säugetierfauna Österreichs.

[78] Jentzsch M., Trost M. (2008). Zum Vorkommen der Gartenspitzmaus *Crocidura suaveolens* (Pallas, 1811) in Sachsen-Anhalt. Hercynia.

[79] Schmidt A. (1998). Reaktionen von Säugetierarten auf die Klimaerwärmung—Eine Auswahl von Beispielen, insbesondere aus der Fledermausfauna. Nyctalus (N.F.).

[80] Schmidt A. (1998). Zur Verbreitungsgeschichte der Gartenspitzmaus *Crocidura suaveolens* in Ostdeutschland. Nat. Und Landsch. Brandenbg..

[81] Vogel P., Cosson J.-F., López Jurado L.F. (2003). Taxonomic status and origin of the shrews (Soricidae) from the Canary islands inferred from a mtDNA comparison with the European *Crocidura* species. Mol. Phylogenet. Evol..

[82] Jaquiéry J., Guélat J., Broquet T., Berset-Brändli L., Pellegrini E., Moresi R., Hirzel A.H., Perrin N. (2008). Habitat-quality effects on metapopulation dynamics in greater white-toothed shrews, *Crocidura russula*. Ecology.

[83] Torten M., Eliash Z., Lawrence D., Shenberg E. (1972). *Crocidura russula*, a hitherto unknown carrier of leptospires. Isr. J. Med. Sci..

[84] Obiegala A., Albrecht C., Dafalla M., Drewes S., Oltersdorf C., Turni H., Imholt C., Jacob J., Wagner-Wiening C., Ulrich R.G. (2017). *Leptospira* spp. in Small Mammals from Areas with Low and High Human Hantavirus Incidences in South-West Germany. Vector Borne Zoonotic Dis..

[85] Obiegala A., Woll D., Karnath C., Silaghi C., Schex S., Eßbauer S., Pfeffer M. (2016). Prevalence and Genotype Allocation of Pathogenic *Leptospira* Species in Small Mammals from Various Habitat Types in Germany. PLoS Negl. Trop. Dis..

[86] Wright T.F., Eberhard J.R., Hobson E.A., Avery M.L., Russello M.A. (2010). Behavioral flexibility and species invasions: The adaptive flexibility hypothesis. Ethol. Ecol. Evol..

[87] Ferreira A.S., Ahmed A., Rocha T., Vieira M.L., Paiva-Cardoso M.d.N., Mesquita J.R., van der Linden H., Goris M., Thompson G., Hartskeerl R.A. (2020). Genetic diversity of pathogenic leptospires from wild, domestic and captive host species in Portugal. Transbound. Emerg. Dis..

[88] Stritof Majetic Z., Galloway R., Ruzic Sabljic E., Milas Z., Mojcec Perko V., Habus J., Margaletic J., Pernar R., Turk N. (2014). Epizootiological survey of small mammals as *Leptospira* spp. reservoirs in Eastern Croatia. Acta Trop..

[89] Renaud C., Andrews S., Djelouadji Z., Lecheval S., Corrao-Revol N., Buff S., Demont P., Kodjo A. (2013). Prevalence of the *Leptospira* serovars *bratislava*, *grippotyphosa*, *mozdok* and *pomona* in French dogs. Vet. J..

[90] Da Cunha C.E.P., Felix S.R., Neto A.C.P.S., Campello-Felix A., Kremer F.S., Monte L.G., Amaral M.G., de Oliveira Nobre M., Da Silva É.F., Hartleben C.P. (2016). Infection with *Leptospira kirschneri* Serovar Mozdok: First Report from the Southern Hemisphere. Am. J. Trop. Med. Hyg..

[91] Jolley K.A., Bray J.E., Maiden M.C.J. Open-Access Bacterial Population Genomics: BIGSdb Software. Wellcome Open Res. 2018, Volume 3. The PubMLST.org Website and Their Applications. https://pubmlst.org/bigsdb?page=info&db=pubmlst_leptospira_isolates&id=503.

[92] Barandika J.F., Hurtado A., García-Esteban C., Gil H., Escudero R., Barral M., Jado I., Juste R.A., Anda P., García-Pérez A.L. (2007). Tick-borne zoonotic bacteria in wild and domestic small mammals in northern Spain. Appl. Environ. Microbiol..

[93] Silaghi C., Beck R., Oteo J.A., Pfeffer M., Sprong H. (2016). Neoehrlichiosis: An emerging tick-borne zoonosis caused by *Candidatus* Neoehrlichia mikurensis. Exp. Appl. Acarol..

[94] Bray D.P., Bown K.J., Stockley P., Hurst J.L., Bennett M., Birtles R.J. (2007). Haemoparasites of common shrews (*Sorex araneus*) in Northwest England. Parasitology.

[95] Lin J.-W., Hsu Y.-M., Chomel B.B., Lin L.-K., Pei J.-C., Wu S.-H., Chang C.-C. (2012). Identification of novel *Bartonella* spp. in bats and evidence of Asian gray shrew as a new potential reservoir of *Bartonella*. Vet. Microbiol..

[96] Liyai R., Kimita G., Masakhwe C., Abuom D., Mutai B., Onyango D.M., Waitumbi J. (2021). The spleen bacteriome of wild rodents and shrews from Marigat, Baringo County, Kenya. PeerJ.

[97] Celebi B., Anani H., Zgheib R., Carhan A., Raoult D., Fournier P.-E. (2021). Genomic Characterization of the Novel *Bartonella refiksaydamii* sp. Isolated from the Blood of a *Crocidura suaveolens* (Pallas, 1811). Vector Borne Zoonotic Dis..

[98] Vieira-Damiani G., Diniz P.P.V.d.P., Pitassi L.H.U., Sowy S., Scorpio D.G., Lania B.G., Drummond M.R., Soares T.C.B., Barjas-Castro M.d.L., Breitschwerdt E.B. (2014). *Bartonella clarridgeiae* Bacteremia Detected in an Asymptomatic Blood Donor. J. Clin. Microbiol..

[99] Špitalská E., Boldiš V., Mošanský L., Sparagano O., Stanko M. (2015). *Rickettsia* species in fleas collected from small mammals in Slovakia. Parasitol. Res..

[100] Morrone J.J., Acosta R. (2006). A synopsis of the fleas (Insecta: Siphonaptera) parasitizing New World species of Soricidae (Mammalia: Insectivora). Zootaxa.

[101] Heglasová I., Rudenko N., Golovchenko M., Zubriková D., Miklisová D., Stanko M. (2020). Ticks, fleas and rodent-hosts analyzed for the presence of *Borrelia miyamotoi* in Slovakia: The first record of *Borrelia miyamotoi* in a *Haemaphysalis inermis* tick. Ticks Tick Borne Dis..

